# Mitigation of acute lung injury by human bronchial epithelial cell-derived extracellular vesicles via ANXA1-mediated FPR signaling

**DOI:** 10.1038/s42003-024-06197-3

**Published:** 2024-05-06

**Authors:** Yu Fujita, Tsukasa Kadota, Reika Kaneko, Yuta Hirano, Shota Fujimoto, Naoaki Watanabe, Ryusuke Kizawa, Takashi Ohtsuka, Kazuyoshi Kuwano, Takahiro Ochiya, Jun Araya

**Affiliations:** 1 Division of Respiratory Diseases, Department of Internal Medicine, The Jikei University School of Medicine, Tokyo, Japan; 2 Division of Next-Generation Drug Development, Research Center for Medical Sciences, The Jikei University School of Medicine, Tokyo, Japan; 3 Center for Exosome Medical Research, The Jikei University School of Medicine, Tokyo, Japan; 4 Division of Thoracic Surgery, Department of Surgery, The Jikei University School of Medicine, Tokyo, Japan; 5 Department of Molecular and Cellular Medicine, Institute of Medical Science, Tokyo Medical University, Tokyo, Japan

**Keywords:** Molecular medicine, Extracellular signalling molecules, Sepsis, Respiratory distress syndrome

## Abstract

Acute lung injury (ALI) is characterized by respiratory failure resulting from the disruption of the epithelial and endothelial barriers as well as immune system. In this study, we evaluated the therapeutic potential of airway epithelial cell-derived extracellular vesicles (EVs) in maintaining lung homeostasis. We isolated human bronchial epithelial cell-derived EVs (HBEC-EVs), which endogenously express various immune-related surface markers and investigated their immunomodulatory potential in ALI. In ALI cellular models, HBEC-EVs demonstrated immunosuppressive effects by reducing the secretion of proinflammatory cytokines in both THP-1 macrophages and HBECs. Mechanistically, these effects were partially ascribed to nine of the top 10 miRNAs enriched in HBEC-EVs, governing toll-like receptor-NF-κB signaling pathways. Proteomic analysis revealed the presence of proteins in HBEC-EVs involved in WNT and NF-κB signaling pathways, pivotal in inflammation regulation. ANXA1, a constituent of HBEC-EVs, interacts with formyl peptide receptor (FPR)2, eliciting anti-inflammatory responses by suppressing NF-κB signaling in inflamed epithelium, including type II alveolar epithelial cells. In a mouse model of ALI, intratracheal administration of HBEC-EVs reduced lung injury, inflammatory cell infiltration, and cytokine levels. Collectively, these findings suggest the therapeutic potential of HBEC-EVs, through their miRNAs and ANXA1 cargo, in mitigating lung injury and inflammation in ALI patients.

## Abstract (web summary)

Human bronchial epithelial cell-derived EVs exhibit immunomodulatory properties through miRNAs & ANXA1, which can suppress NF-κB signaling. The study holds promise as a novel approach for mitigating lung injury and inflammation in acute lung injury.

## Introduction

Acute lung injury (ALI) is a prevalent pulmonary condition characterized by respiratory failure stemming from the disruption of the lung epithelial and endothelial barriers. This disruption leads to the flooding of the alveolar compartment with protein-rich fluid and the recruitment of neutrophils into the alveolar space^1^. ALI can be triggered by various insults, including direct lung causes such as bacterial or viral pneumonia, aspiration, or extrapulmonary factors such as sepsis or trauma. Histologically, ALI is marked by an acute pulmonary inflammatory response that severely impairs oxygenation capacity. Recent research has illuminated how the excessive activation of inflammatory cells, including monocytes and macrophages, can contribute to adverse lung inflammatory responses, ultimately culminating in ALI^2^. Indeed, alveolar macrophages, the primary immune cells residing in the lung tissue, can exert either pro-inflammatory or anti-inflammatory effects depending on the specific microenvironment during various stages of ALI. Importantly, the cells involved in the regulation of lung injury and their mechanisms may differ in various ALI models due to different underlying causes. For instance, in the case of SARS-CoV-2 infection, a pathogenic stimulus directly affecting lung epithelial cells triggers an innate immune response, initiating acute lung inflammation^3^. Conversely, in extrapulmonary ALI models, injury to the lung microvascular endothelial cells represents the earliest cellular event leading to interstitial edema^4^. A comprehensive understanding of the mechanisms involved in different ALI models is essential for determining effective strategies to prevent ALI development and improve patient prognosis. Despite advancements in our understanding of ALI pathophysiology, the efficacy of standard treatments, such as lung-protective ventilation, prone positioning, and neuromuscular blockers, is frequently limited. Therefore, a compelling necessity exists to develop novel therapeutic strategies to effectively mitigate pulmonary inflammation and decrease lung injury.

Stem cell-based therapies have gained increasing interest as novel treatments for ALI^5^. Among these, mesenchymal stem cells (MSCs), found in various adult tissues, have garnered significant attention owing to their multipotent differentiation capability. Studies have demonstrated that MSCs effectively modulate host immune responses and facilitate tissue repair following lung injuries^6^. Type II alveolar epithelial cells (ATII) function as local unipotent stem cells responsible for repairing the alveolar epithelium during both steady-state replacement and after injury^7^. Notably, experiments involving the transplantation of embryonic stem cell-derived ATII cells into ALI-affected mice have yielded promising results, leading to improved lung injury outcomes, including body weight recovery, enhanced arterial blood oxygen saturation, reduced collagen deposition, and increased survival rates^8^. Hence, both MSCs and lung epithelial cells show promise as candidates for innovative ALI treatments. Recent research also suggests that the therapeutic effects of stem cells are attributed to the release of substances such as cytokines, growth factors, and extracellular vesicles (EVs)^9^.

EVs are lipid bilayer-enclosed particles released by all cell types, both in normal and disease conditions^10^. These vesicles serve as carriers of proteins, mRNAs, microRNAs (miRNAs), and lipids from their parent cells, thereby inducing signal transduction in recipient cells. Moreover, whether secreted by lung or non-lung cells, EVs have been found to play a crucial role in regulating both physiological and pathological activities within pulmonary tissues by facilitating alveolar cell crosstalk events in the lung microenvironment^11^. Furthermore, emerging evidence suggests that EVs derived from MSC (MSC-EVs) hold promise as a cell-free therapeutic approach and can effectively function in various tissue injuries, including ALI^12^. The regenerative potential of ATII cells highlights the therapeutic promise of ATII-derived EVs in ALI. However, obtaining sufficient quantities of human ATII cells for isolating EVs for injection therapies remains a significant challenge. Therefore, our focus shifted to airway epithelial cells as a viable source of EVs, with the aim of elucidating their role in lung physiology and pathology^13,14^. Recent data showed that airway epithelial cells can communicate with each other through EVs^15^. As one type of epithelial cells, human bronchial epithelial cells (HBECs) have the potential to maintain lung homeostasis against environmental stimuli. Our research has revealed that EVs derived from healthy human bronchial epithelial cells (HBEC-EVs) efficiently suppress transforming growth factor (TGF)-β-WNT-induced myofibroblast differentiation and lung epithelial cell senescence. The effects of HBEC-EVs in mitigating pulmonary fibrosis were more potent than those of MSC-EVs^14^. Notably, the HBECs express the basal cell markers, such as transformation-related protein 63 (Trp63) and cytokeratin 5 (Krt5)^16^, indicating that HBEC-EVs can contribute to lung injury repair. However, the potential effects of HBEC-EVs in the treatment of acute inflammatory lung diseases have not been thoroughly investigated.

In the present study, we conducted a comprehensive examination of the immunomodulatory mechanisms of HBEC-EVs utilizing cellular models of LPS-induced ALI or poly (I:C). We aimed to gain insights into the intricate immunomodulatory mechanisms governed by HBEC-EVs, with a particular focus on elucidating the role of their miRNA and protein cargo. Additionally, we sought to assess the therapeutic properties of HBEC-EVs in a mouse model of ALI induced by the administration of both LPS and poly (I:C) through intratracheal delivery of these vesicles. Our findings establish a novel theoretical foundation for the targeted treatment of ALI.

## Materials and Methods

### Antibodies and reagents

The following antibodies were used: mouse anti-CD9 (Santa Cruz Biotechnology, sc-59140), mouse anti-CD63 (BD Pharmingen, 556019), mouse anti-CD81 (Santa Cruz Biotechnology, sc-555675), mouse anti-β-actin (Millipore, MAB1501), mouse anti-MHC-2 (Cell Signaling Technology, 68258), mouse anti–NK-κB (Cell Signaling Technology, 8242), mouse anti–phosphor-NK-κB (Cell Signaling Technology, 3033), rabbit anti-ANXA1 (Thermo Fisher Scientific, 71-3400), rabbit anti-FPR2 (Novusbio, NLS1878), mouse anti-HT2-280 (Terrace, TB-27AHT2-280), and rabbit anti-SFTPC (Millipore, AB3786). Additionally, the following reagents were used: the Human MACSPlex Exosome Kit (Miltenyi Biotec, 130-108-813), Phorbol 12-myristiate-12 acetate (PMA Sigma-Aldrich, P8139), poly (I:C) (InvivoGen, tlrl-pic), lipopolysaccharide LPS (Sigma‐Aldrich, L2630), dexamethasone (Fujifilm, 041-18861), recombinant ANXA1 (American Research products, 01-2062), and WRW4 (R&D Systems, 2262/1).

### Cell culture and clinical samples

HBECs were either purchased from Lonza or isolated from normal airway tissue (1st through 4th order bronchi) using protease treatment as previously described^16^. Airway tissues were obtained from pneumonectomy and lobectomy specimens of patients with lung cancer at The Jikei University School of Medicine. Informed consent was obtained from all surgical participants as part of an ongoing research protocol approved by the Ethical Committee of Jikei University School of Medicine (#20‐153 (5443)). All ethical regulations relevant to human research participants were followed. The participants lacked a history of pulmonary fibrosis, COPD or any other inflammatory diseases. The isolated HBECs were plated onto rat tail collagen type I‐coated (10 μg/ml) (Sigma‐Aldrich, C3867) dishes in Bronchial Epithelial Growth Medium (BEGM, Lonza) containing 1% antibiotic–antimycotic solution (Thermo Fisher Scientific) and grown at 37 °C in 5% CO_2_. The HBECs were used until passage five. For the isolation and purification of human ATII cells was performed as described previously^17^. Briefly, a piece of lung tissue was mechanically minced and dissociated with enzymes according to the Lung Dissociation Kit protocol (130-095-927; Miltenyi Biotec). A Miltenyi Biotec gentle MACS dissociator was used for mincing and incubation for 42 min at 37 °C. The cells were washed and passed over 70-μM and 40-μM filters, and red blood cells were lysed with red-blood-cell lysis buffer (42574000; Miltenyi Biotec). We then incubated the cells with anti-HT2-280 antibody and coupled with anti-mouse IgM-coated magnetic beads to allow magnetic bead-based ATII separation. We then used the Miltenyi MACS bead sorting kit to separate ATII according to the manufacturer’s procedure (Miltenyi Biotec, Germany). Subsequently, the cell suspension was cultured on rat tail collagen type I‐coated (10 μg/ml) dishes in Small Airway Epithelial Growth Medium (SAGM, Lonza) containing 1% antibiotic–antimycotic solution (Thermo Fisher Scientific) and grown at 37 °C in 5% CO_2_. To facilitate the differentiation of ATII cells, experiments were initiated promptly after cell seeding. Human monocytic THP-1 cells were obtained from the American Type Culture Collection (ATCC) and were maintained in RPMI 1640 medium with 10% heat-inactivated fetal bovine serum and an antibiotic-antimycotic at 37 °C in 5% CO_2_. THP-1 cells were differentiated with PMA for the experiments.

### Cell transfection

For miRNA transfection at 25 nM, Lipofectamine 3000 reagent (Thermo Fisher Scientific) was used according to the manufacturer’s protocol. HBEC or THP-1 cells were seeded at 50% confluence the day before transfection. The miRNA mimics used for transfection included miR-7-5p, miR-125b-5p, let-7a-5p, miR-125a-5p, let-7b-5p, let-7f-5p, let-7i-5p, miR-26a-5p, miR-16-5p, let-7c-5p, and negative control miRNA (miR-NC) (Thermo Fisher Scientific).

### EV purification by ultracentrifugation

After reaching 50% confluence on 10 cm‐cell culture dishes, HBECs for EV preparation were washed with PBS. Fresh BEGM containing 1% antibiotic-antimycotics replaced the culture medium. After 48 h of incubation, conditioned medium (CM) was collected and centrifuged at 2000 g for 10 min at 4 °C. The resulting supernatant was then filtered through a 0.22 μm filter (Millipore) to completely remove cellular debris. For EV preparation, the CM underwent ultracentrifugation at 4 °C for either 70 min at 35,000 rpm using an SW41Ti rotor or 45 min at 44,200 rpm using an MLS50 rotor. The resulting pellets were washed by ultracentrifugation and resuspended in PBS. Protein concentrations of the putative EV fractions were measured using a Quant‐iT Protein Assay and a Qubit 2.0 Fluorometer (Thermo Fisher Scientific). To determine the size distribution of the EVs, nanoparticle tracking analysis was performed using the Nanosight system (NanoSight LM300) with samples diluted 500‐fold with PBS.

### Phase contract electron microscopy

The isolated HBEC EVs were examined using a phase-contrast transmission electron microscope (Terabase Inc.). This imaging technique allows for the generation of high-contrast images of the nanostructures of soft materials without the need for staining processes that might damage the samples. The natural structure of the EV sample distributed in solution was observed by preparing the sample using a rapid vitreous ice‐embedding method and cryo‐phase‐contrast transmission electron microscopy, as previously described^18^.

### RNA extraction and qRT-PCR

Total RNA was extracted from cultured cells or EVs using QIAzol and the miRNeasy Mini Kit (Qiagen), following the manufacturer’s protocol. The purity and concentration of all RNA samples were quantified using a NanoDrop ND-1000 spectrophotometer (Thermo Fisher Scientific) or an Agilent 2100 Bioanalyzer (Agilent). Reverse transcription was performed using the High-Capacity cDNA Reverse Transcription Kit (Thermo Fisher Scientific) with random hexamer primers. The synthesized cDNAs were quantified using SYBR Green I qRT-PCR. qRT-PCR analysis was conducted using primers designed with PrimerBank (https://pga.mgh.harvard.edu/primerbank/). β-actin was utilized for normalization. Supplementary Table 2 shows the sequences of the primers in this study. The relative expression of each gene was measured using the 2(- Delta C(T)) method. Reactions were performed using the QuantStudio^TM^ 3 Real-Time PCR System (Thermo Fisher Scientific). All reactions were performed in triplicate.

### Western blot analysis

Cultured cells, EVs, and mouse lungs were lysed in a mammalian protein extraction reagent (M-PER; Thermo Fisher Scientific) using a sample buffer solution (Wako, 198-13282 or 191-13272). For each experiment, equal amounts of total protein were loaded onto a 4%–20% SDS-PAGE gel (Bio-Rad). Following SDS-PAGE, proteins were transferred to a polyvinylidene difluoride membrane (Millipore). After blocking with Blocking One (Nacalai Tesque), membranes were incubated with a specific primary antibody for 1 h at room temperature. Secondary antibodies (anti-rabbit IgG Cell Signaling Technology, 5127) and anti-mouse IgG (GE Healthcare, NA931) were used at a dilution of 1:1,000. The membranes were then subjected to chemiluminescence detection (Thermo Fisher Scientific, 34080) using a ChemiDoc^TM^ Touch Imaging System (Bio-Rad).

### ELISA

Human IL-6 (R&D Systems, D6050) levels in culture supernatants were determined by ELISA according to the manufacturers’ protocols. Concentrations were determined by comparison with standard curves.

### Immunofluorescence staining

After washing three times with PBS, cells were fixed in 4% paraformaldehyde (Wako), and incubated for 1 hour with primary antibodies containing 0.1% BSA. The cells were then incubated with Alexa Fluor fluorescent secondary antibodies containing 0.1% BSA. Hoechst33342 staining was performed immediately before imaging. All staining was observed using a confocal microscope (LSM 880, ZEISS).

### Animal studies

All animal experiments performed in this study were approved by the Institute for Laboratory Animal Research, Jikei University School of Medicine (Number: 2018–071). Male mice aged 6–7 weeks C57BL/6 J (Charles River Laboratories) were employed in the experiments. Briefly, LPS was intraperitoneally (i.p.) injected at a dose of 5 μg/mg in 50 μl PBS. Four hours following LPS injection, poly (I:C) at a dose of 5 μg/mg in 50 μl PBS and HBEC-EVs (2 × 10^9^ particles)/PBS control was intratracheally (i.t.) injected. After 24 h of induction, the mice were euthanized. Lung tissue sections were stained with hematoxylin-eosin (HE). The lung inflammation score was calculated using a previously reported method^19^. BALF was collected by cannulating the trachea with a 20-gauge shielded intravenous catheter, instilling 0.8 ml of sterile PBS four times, and collecting the fluid by gentle aspiration. The BALF was centrifuged for 5 min at 400 × g on microscopic slides, and the BALF cells were stained using the Diff-Quick method. The resulting fluid was passed through a 0.45-μm filter and either used immediately or stored at −70 °C for subsequent measurements of IL-6 and TNF-α levels by ELISA (R&D Systems). We have complied with all relevant ethical regulations for animal use.

### Immunohistochemistry staining

Lung samples were initially fixed in formalin and embedded in paraffin. Following the dewaxing and rehydration process, heat-induced epitope retrieval was performed by immersing the specimens in 1/200 diluted ImmunoSaver solution (Nissin EM, Tokyo, Japan) at 98 °C for 45 min. Endogenous peroxidases were inactivated by treating the specimens with 3% H_2_O_2_ at room temperature for 10 min. The specimens were then permeabilized with 0.1% Triton X-100. After treatment with a protein block serum-free blocking reagent (DAKO, X0909) at room temperature for 30 min, the specimens were incubated with the primary antibody at room temperature for 1 h or at 4 °C overnight. The sections were stained using ImPRESS IgG-peroxidase kits (Vector Laboratories) and a metal-enhanced DAB substrate kit (Life Technologies), following the manufacturer’s instructions. After counterstaining with hematoxylin, the specimens were dehydrated and mounted.

### Statistics and reproducibility

The data presented in bar graphs are displayed as the average ( ± SEM) for technical replicates. Analysis of variance (ANOVA) was employed for multiple comparisons, followed by Tukey’s or Dunnett’s multiple comparisons to evaluate the differences. Statistical significance was defined as *P* < 0.05. Prism version 9 (GraphPad Software, San Diego, CA, USA) was utilized for statistical analysis. For gene ontology (GO) enrichment analyses, we used DIANA-mirPath^20^ or DAVID 2021 (https://david.ncifcrf.gov). All data were obtained from three independent replicates to mitigate the influence of chance.

### Reporting summary

Further information on research design is available in the Nature Portfolio Reporting Summary linked to this article.

## Results

### Characterization of HBEC-EVs

**Fig. 1:**
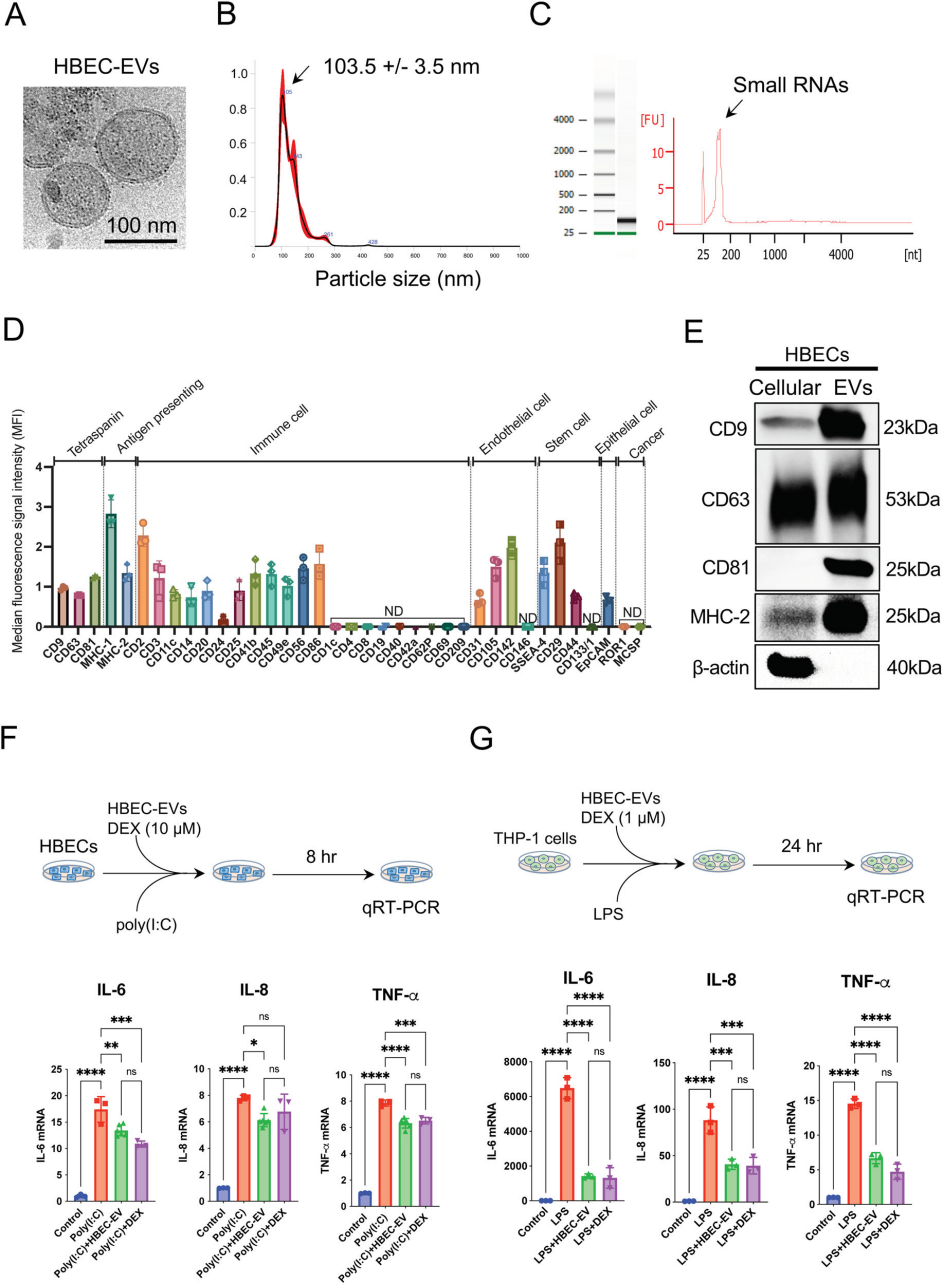
Characterization and functions of HBEC-EVs. (**A**) Cryotransmission electron microscopy of purified HBEC-EVs using ultracentrifugation. (**B**) Nanoparticle tracking analysis displaying the particle size of purified HBEC-EVs. The vertical axis in the graph represents the number of EV particles (x 10^8^)/mL, and the horizontal axis indicates the particle size (nm) of EVs. (**C**) Analysis of HBEC-EVs using a bioanalyzer. Gels and electropherogram are presented. The left gel lane is the ladder standard, and the right lane contains the total RNA from HBEC-EVs. The *y*-axis of the electropherogram displays signal intensities in arbitrary fluorescence units (FU), and the *x*-axis indicates the size of the RNA in nucleotides (nt). (**D**) Utilization of a multiplex bead-based flow cytometry assay to detect HBEC-EV surface signatures. Thirty-seven multiplexed populations of dye-labeled antibody-coated capture beads were incubated with three different HBEC-EV samples. The mean fluorescence intensity (MFI) of each marker was analyzed. ND: not detected. (**E**) Western blots of HBECs and HBEC-EVs for CD9, CD63, CD81, MHC-2, and β-actin. (**F-G**) Measurement of cytokine secretion in HBECs at 8 h after poly (I:C) (250 ng/ml) and HBEC-EV administration (10 μg/ml) (**F**) and in THP-1 cells at 24 h after LPS (1 μg/ml) and HBEC-EV administration (10 μg/ml) (**G**). THP-1 cells were treated with 100 nM PMA for 24 h before the experiments. ****P < 0.0001, ***P < 0.001, **P < 0.01, *P < 0.05. NS; not significant.

HBEC-EVs were isolated through conventional ultracentrifugation of conditioned medium obtained from primary HBECs and underwent extensive characterization. Cryotransmission electron microscopic analysis revealed the presence of typical bilayer membrane vesicles, displaying heterogeneity in size (Fig. 1A). NanoSight tracking analysis indicated that HBEC-EVs had an average diameter of approximately 100 nm (Fig. 1B). When we assessed the RNA content of HBEC-EVs, we observed that the RNA profile of HBEC-EVs contained only minimal amounts of ribosomal RNA but substantial amounts of small RNAs (Fig. 1C). To comprehensively profile the surface markers of HBEC-EVs, we performed multiplex bead-based cytometry, enabling the simultaneous detection and semi-quantitative analysis of 37 distinct EV surface epitopes. In total, 24 out of 37 markers (64.9%) were detected in HBEC-EVs. These surface markers encompassed various categories, including tetraspanins (CD9, CD63, and CD81), antigen-presenting proteins (MHC-1, MHC-2), immune cell markers (CD2, CD3, CD11c, CD14, CD20, CD24, CD25, CD41b, CD45, CD49e, CD56, CD86), endothelial cell markers (CD31, CD105, CD142), MSC markers (SSEA-4, CD29, CD44), and an epithelial cell marker (EpCAM) (Fig. 1D). The data indicated the presence of several immune-related markers, mostly at low to intermediate positive intensity levels. Notably, MHC-1 exhibited the highest intensity, suggesting the potential immunomodulatory role of HBEC-EVs. Furthermore, western blot analysis confirmed the presence of EV marker proteins, including CD9, CD63, CD81, and MHC-2, whereas the actin cytoskeleton was not enriched in the EVs (Fig. 1E). These findings align with the minimal experimental criteria established for identifying small EVs, as outlined in the position statement of the International Society for EVs^21^.

### Immunomodulatory capacity of HBEC-EVs in cellular models of ALI

Lung epithelial cells respond to similar injuries and infections by regulating leukocyte influx through the production of pro-inflammatory cytokines and chemokines^22^. To simulate a proinflammatory epithelium, we assessed HBECs after the addition of HBEC-EVs and poly (I:C). Notably, the introduction of poly (I:C) in HBECs led to increased expression levels of pro-inflammatory cytokines, including IL-6, IL-8, and TNF-α. However, this response was mitigated by HBEC-EV treatment (Fig. 1F). In contrast, several immune cells, including alveolar macrophages, play essential roles in initiating immune cascades that contribute to ALI development^23^. To replicate immune activation in ALI, PMA-induced-induced THP-1 macrophages were treated with poly (I:C) and HBEC-EVs. In this experimental context, poly(I:C) was unable to elicit an adequate production of pro-inflammatory cytokines in THP-1 cells. As a result, we explored the use of LPS as a stimulator. Exposure to LPS in THP-1 cells increased expression levels of pro-inflammatory cytokine such as IL-6, IL-8, and TNF-α, whereas HBEC-EV treatment attenuated these elevated levels (Fig. 1G). The immunosuppressive capacity of HBEC-EVs in the THP-1 cells was comparable with that of 1 μM dexamethasone (DEX) treatment. The immunosuppressive potential of HBEC-EVs matched that of 10 μM DEX. These findings highlight the immunomodulatory role of HBEC-EVs during and after the onset of lung inflammation.

### Impact of HBEC-EV miRNAs on immunomodulation

**Fig. 2:**
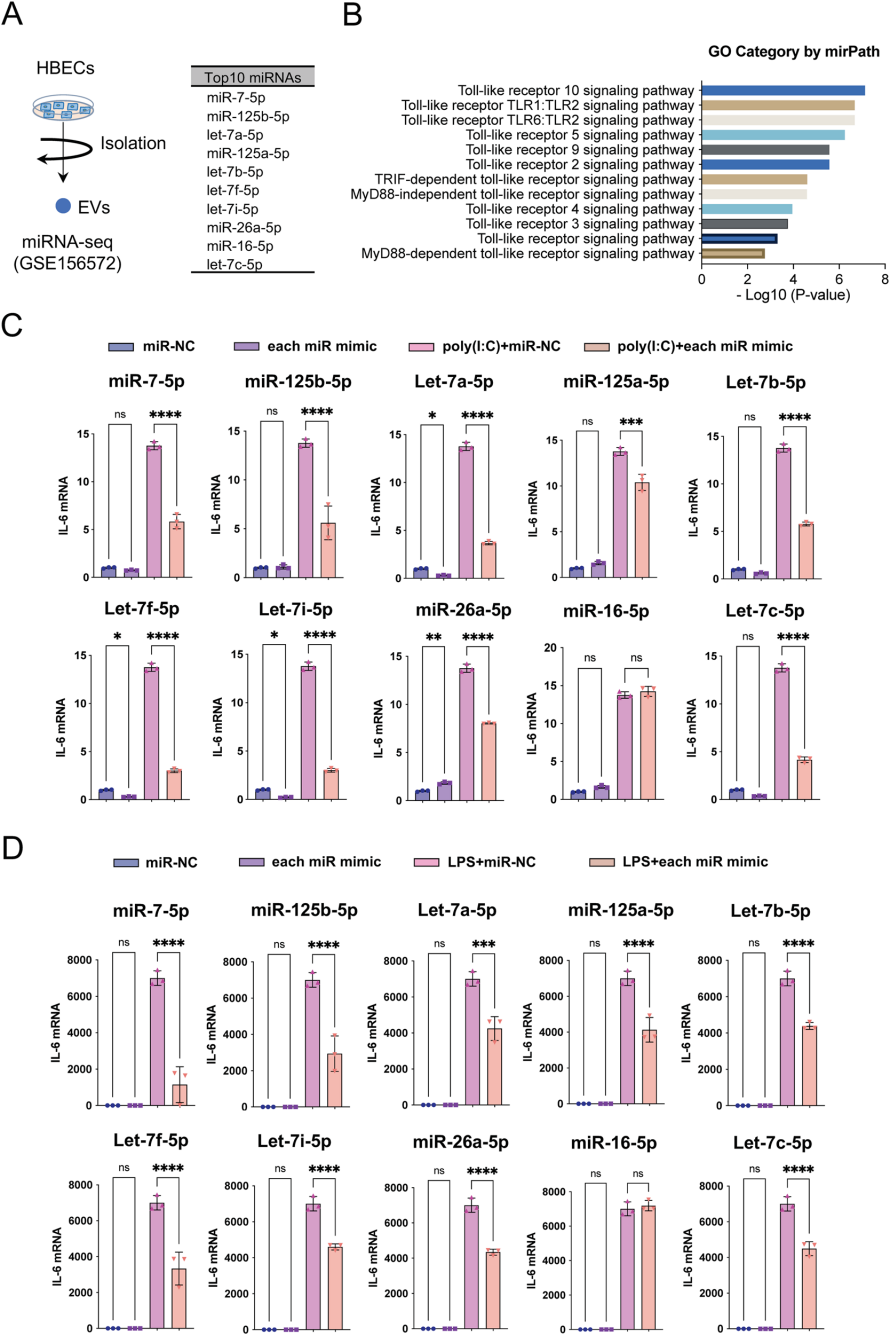
The immunomodulatory capacity of HBEC-EV miRNAs. (**A**) Expression profiles of miRNAs in HBEC-EVs obtained through miRNA-seq analysis (GSE156572)^14^. The top 10 miRNAs enriched in HBEC-EVs listed in descending order of expression. (**B**) Gene ontology analysis from DIANA-mirPath v3.0 analysis displaying the 12 significant immune responses pathways among the top 50 pathways, based on the 10 most abundant miRNAs in HBEC-EVs. (**C**) IL-6 mRNA expression in HBECs at 8 h after poly (I:C) exposure (250 ng/ml) and each pre-miRNA transfection. ****P < 0.0001, ***P < 0.001, **P < 0.01, *P < 0.05. NS; not significant. (**D**) IL-6 mRNA expression of THP-1 cells at 24 h after LPS exposure (1 μg/ml) and each pre-miRNA transfection. THP-1 cells were treated with 100 nM PMA for 24 hours before the experiments. ****P < 0.0001, ***P < 0.001. NS; not significant.

To uncover the underlying mechanism, we delved into the composition of HBEC-EVs. The EV-mediated transfer of miRNAs has been established as a means to regulate gene expression in recipient cells, thereby modulating their function^24^. In a prior study, we reported the miRNA expression profiles in HBEC-EVs using miRNA-seq analysis (GSE156572)^14^. Consequently, our focus turned to the top 10 miRNAs encapsulated in HBEC-EVs, which include miR-7-5p, miR-125b-5p, let-7a-5p, miR-125a-5p, let-7b-5p, let-7f-5p, let-7i-5p, miR-26a-5p, miR-16-5p, and let-7c-5p (listed in the descending order of expression) (Fig. 2A). To understand the biological functions of these top 10 miRNAs within HBEV-EVs, we conducted GO analyses using DIANA-mirPath, which revealed that 12 of the top 50 pathways were linked to toll-like receptor (TLR) signaling pathways (Fig. 2B). Further GO analysis highlighted a significant enrichment of miRNAs associated with the regulation of immune responses and various inflammatory pathways.

Subsequently, we evaluated IL-6 mRNA expression in HBECs after exposure to poly (I:C) and pre-miRNA transfection. Indeed, we confirmed that the overexpression of these same nine miRNAs significantly decreased IL-6 expression in HBECs treated with poly (I:C) (Fig. 2C). Next, we examined IL-6 mRNA expression in PMA-induced THP-1 macrophages after LPS exposure and pre-miRNA transfection. The qRT-PCR results demonstrated that overexpression of pre-miR-7-5p, -miR-125b-5p -let-7a-5p, -miR-125a-5p, -let-7b-5p, -let-7f-5p, -let-7i-5p, -miR-26a-5p, and -let-7c-5p (excluding -miR-16-5p) significantly decreased IL-6 expression in THP-1 cells treated with LPS (Fig. 2D). Our study unveiled that nine out of the top 10 miRNAs found in HBEC-EVs played a role in inhibiting the secretion of pro-inflammatory cytokines in in vitro models of ALI. Collectively, these findings suggest that miRNAs enriched in HBEC-EVs may contribute to the reduction of lung injury and inflammation.

### Proteomic analysis of HBEC-EVs and immunomodulation via EV protein cargo

**Fig. 3:**
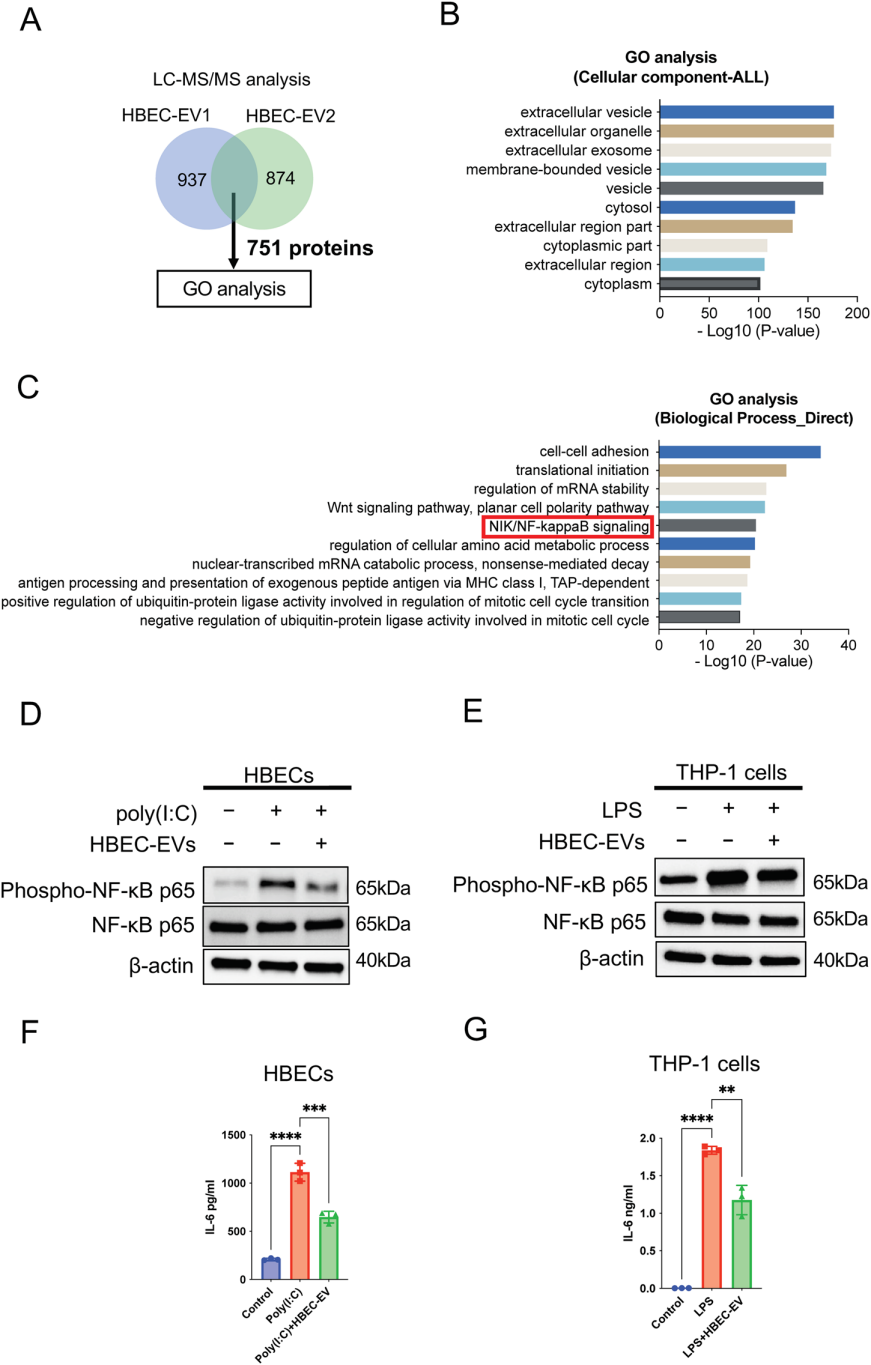
The immunomodulatory capacity of HBEC-EV proteins. (**A**) Venn diagrams illustrating the overlap between proteins from two different HBEC-EVs. (**B**) GO analysis concerning cellular component_ALL from DAVID 2021 of proteins co-expressed in two different HBEC-EVs. (**C**) GO analysis related to biological process_Direct from DAVID 2021 of proteins co-expressed in two different HBEC-EVs. (**D-E**) Western blots showing the amount of p-NF-κB, NF-κB, and β-actin in HBECs (**D**) or THP-1 cells (**E**) treated with HBEC-EVs in the presence of poly (I:C) (250 ng/ml) (**D**) or LPS (1 μg/ml) (**E**). (**F-G**) ELISA showing the amount of IL-6 protein levels in HBECs (**F**) or THP-1 cells (**G**) treated with HBEC-EVs in the presence of poly (I:C) (250 ng/ml) (**F**) or LPS (1 μg/ml) (**G**). THP-1 cells were treated with 100 nM PMA for 24 h before the experiments.

Next, we conducted a proteomic analysis of HBEC-EVs using liquid chromatography-mass spectrometry (LC-MS/MS) for a better understanding. Our previous study had already reported the expression profiles of proteins in HBEC-EVs analyzed using LC-MS/MS^14^. To elucidate the biological roles of the proteins within HBEC-EVs, we utilized DAVID 2021 for gene ontology (GO) enrichment analysis, focusing on the 751 overlapping proteins found in the two distinct HBEC-EVs (Fig. 3A). In alignment with EV characteristics, the GO analysis of cellular components demonstrated that the majority of the identified proteins were exosome-related and membranous (Fig. 3B). Furthermore, the biological process analysis indicated that the HBEC-EV cargo was significantly enriched for proteins responsible for cell-cell adhesion, translational initiation, and the regulation of mRNA stability. Remarkably, we observed enrichment of proteins related to WNT signaling and NF-κB signaling pathways among the top 10 biological processes identified in the GO analysis (Fig. 3C). Our previous research had already established that HBEC-EVs possess therapeutic anti-fibrotic properties through the inhibition of WNT signaling through miRNA cargo, which is effective in preventing the development of pulmonary fibrosis^14^. To clarify novel molecular mechanisms underlying HBEC-EV-mediated immunomodulation, we examined alterations in NF-κB signaling pathways in response to EV treatment in HBECs and THP-1 cells. As previously reported, poly (I:C) or LPS treatment resulting in evident activation of NF-κB signaling, as indicated by p-NF-κB expression. Furthermore, HBEC-EV treatment markedly reduced the levels of p-NF-κB and IL-6 proteins in poly (I:C)-treated HBECs (Fig. 3D&F) as well as in LPS-treated THP-1 cells (Fig. 3E&G).

Based on these data, we examined the top 10 miRNAs present in HBEC-EVs that are involved in toll-like receptor (TLR) signaling, as all TLR singling pathways ultimately lead to the activation of NF-κB pathways. Previous studies have shown that these top 10 miRNAs directly target various NF-κB pathway-related genes in humans and mice (Supplementary Table 1, Supplementary Fig. 1). These findings suggest the involvement of the TLR-NF-κB signaling pathway in the mechanisms responsible for HBEC-EV-mediated immunomodulation through proteins and miRNA cargo.

### HBEC-EV-mediated modulation of NF-κB pathway through ANXA1/FPR signaling

**Fig. 4:**
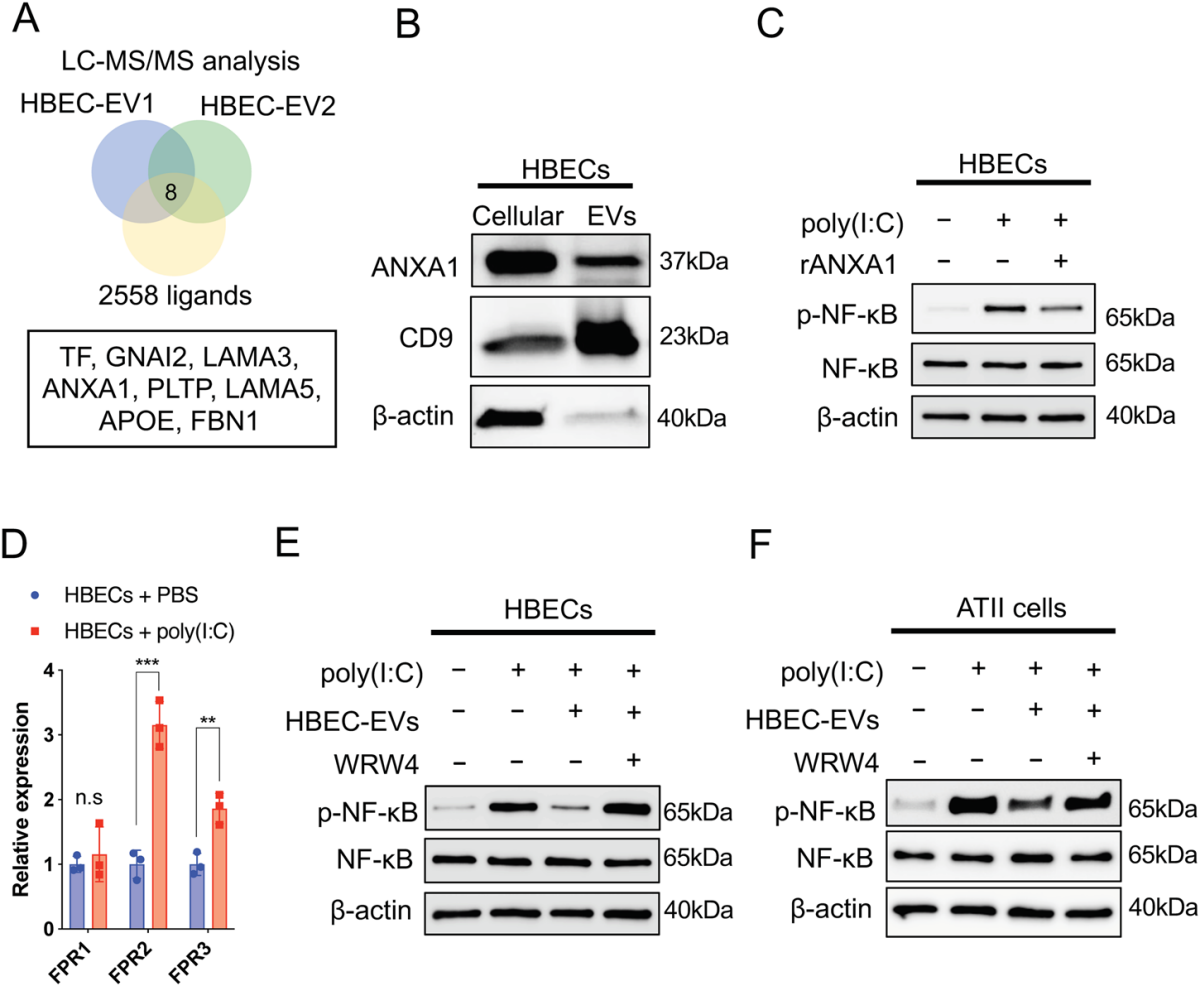
The immunomodulatory capacity of HBEC-EVs through ANXA1-FPR2 signaling in HBECs. (**A**) Venn diagrams displaying overlapping ligands between 751 EV surface proteins from two different HBEC-EVs and 2558 ligands proteins from the FANTOM5 tool. (**B**) Western blots of HBECs and HBEC-EVs for ANXA1, CD9, and β-actin. (**C**) Western blots showing the amount of p-NF-κB, NF-κB, and β-actin in HBECs treated with recombinant ANXA1 (rANXA1) in the presence of poly (I:C) (250 ng/ml). (**D**) FPR1, FPR2, and FPR3 mRNA expression in HBECs at 24 h after poly (I:C) exposure (250 ng/ml). ***P < 0.001, **P < 0.01. NS; not significant. (**E-F**) Western blots showing the amount of p-NF-κB, NF-κB, and β-actin in HBECs (**E**) or type II alveolar epithelial cells (ATII) cells (**F**) treated with HBEC-EVs in the presence of poly (I:C) (50 ng/ml) and WRW4 (10 μM).

To gain a deeper understanding of how HBEC-EVs mediate immunomodulation through the NF-κB signaling, we focused on the interaction between the ligands on the EVs and the receptors on recipient cells. An interactive visualization and query tool for studying ligand-receptor networks in humans has been previously reported (http://fantom.gsc.riken.jp/5/suppl/Ramilowski_et_al_2015/)^25^. Using the FANTOM5 tool, we identified eight common ligands: transferrin (TF), guanine nucleotide binding protein, alpha inhibiting activity polypeptide 2 (GNAI2), laminin, alpha 3 (LAMA3), annexin A1 (ANXA1), phospholipid transfer protein (PLTP), laminin, alpha 5 (LAMA5), apolipoprotein E (APOE), and fibrillin 1 (FBN1), listed in descending order of expression, from a pool of 751 EV surface proteins and the 2558 ligands (Fig. 4A). Among the eight overlapped ligands, we singled out ANXA1, a protein recognized for its potential to suppress NF-κB signaling ^26^, for further analysis. Furthermore, our western blot analysis confirmed the presence of ANXA1 in the HBEC-EVs (Fig. 4B).

We assessed the role of ANXA1 in HBEC-EV-mediated immunomodulation using recombinant ANXA1 ligand (rANXA1). Our findings indicated that rANXA1 treatment significantly suppressed the expression of p-NF-κB in poly (I:C)-treated HBECs (Fig. 4C). Notably, rANXA1 treatment did not significantly suppress the expression of p-NF-κB in LPS-treated THP-1 cells (Supplementary Fig. 2). In the FANTOM5 tool, ANXA1 interacts with receptors such as dysferlin (DYSF), epidermal growth factor receptor (EGFR), and formyl peptide receptors (FPR)1, FPR2, and FPR3. As FPRs, especially FFR2, are major ligands of ANXA1 in humans and are known for their crucial roles in host defense and regulating of inflammatory responses^27^, we focused on FPRs for as potential receptors of EV ANXA1. ANXA1 primarily binds to mainly FPR2, resulting in anti-inflammatory responses and the suppression of both innate and adaptive immune processes through NF-κB and MAPK pathways^27^. Furthermore, it has been reported that FPR2 is mainly expressed in leucocytes, endothelial cells, and epithelial cells^28^. After treatment with poly (I:C), we observed increased gene expression of FPR2 and FPR3 in HBECs (Fig. 4D). In humans, FPR2 shares a 72% amino acid sequence homology with FPR3^28,29^. Consequently, we focused on investigating the role of EV ANXA1-FPR2 signaling in lung injury and inflammation. Importantly, the blockade of FPR2 using a specific antagonist, WRW4, reversed the HBEC-EV-mediated immunomodulatory effect on NF-κB signaling (Fig. 4E), prompted further investigation. For additional validation of this experiment, we employed ATII cells as the recipient cells for HBEC-EV treatment. Primary ATII cells were isolated from human lung samples, cultured in a 2D environment, and confirmed through fluorescence immunostaining to be HT2-280^+^/SFTPC^+^ cells (Supplementary Fig. 3). Notably, the blockade of FPR2 using WRW4 successfully reversed the HBEC-EV-mediated immunomodulatory effect on NF-κB signaling in the ATII cells (Fig. 4F).

In summary, ANXA1 plays a central role in immunomodulating HBEC-EVs through FPR2-NF-κB signaling in inflamed bronchial and alveolar epithelial cells, at least in this experimental setting.

### HBEC-EVs attenuate ALI in vivo through the FPR-NF-κB signaling pathway

**Fig. 5:**
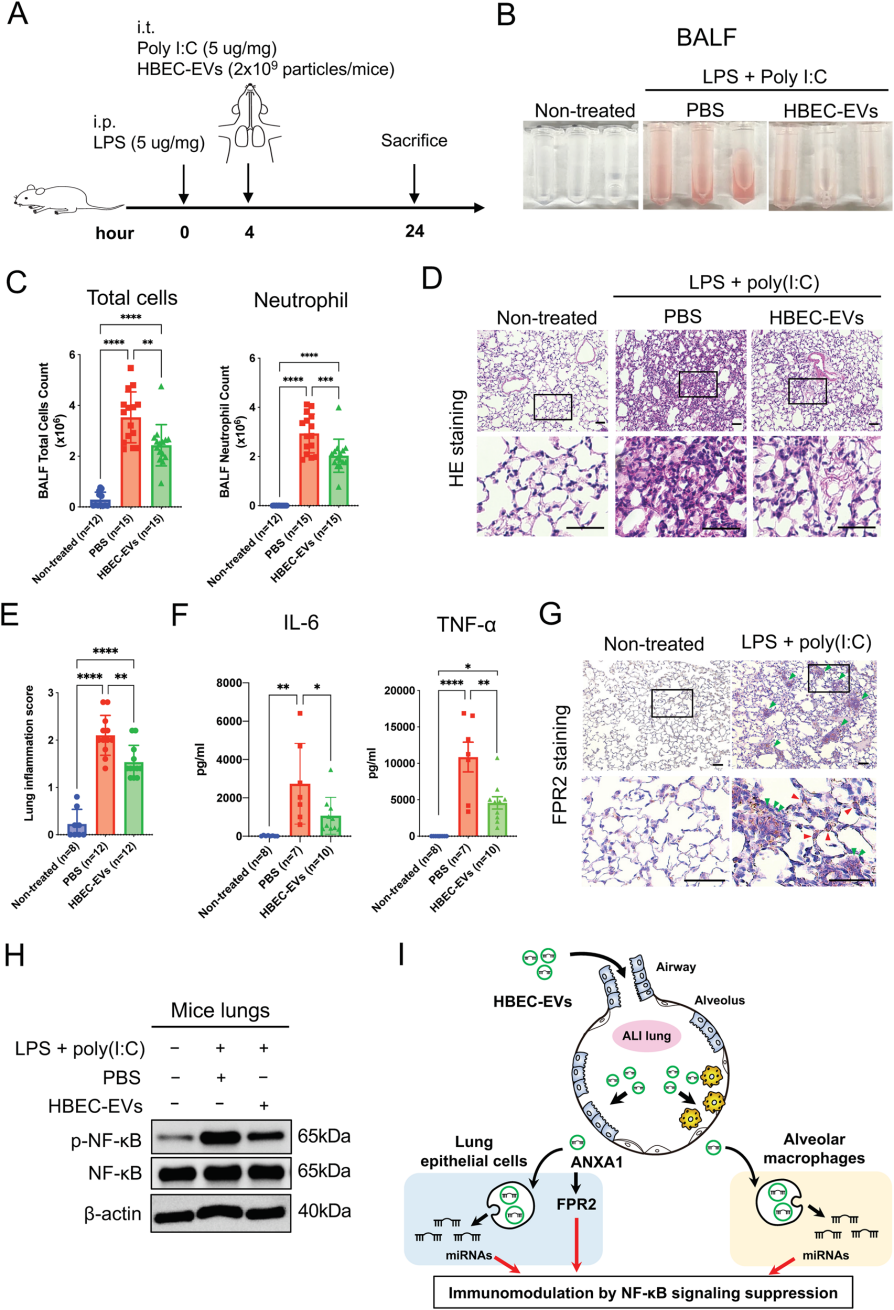
Intratracheal administration of HBEC-EVs attenuate acute lung injury in mice. (**A**) Schematic protocol for intratracheal (i.t.) HBEC-EV treatment in a mouse model of ALI induced by both LPS (intraperitoneal: i.p.) and poly (I:C) (i.t.). (**B**) BALF samples from the ALI model mice. (**C**) The number of total cells and neutrophils in the BALF of the ALI model mice (non-treated *n* = 12, ALI_PBS *n* = 15, ALI_HBEC-EVs *n* = 15). ****P < 0.0001, ***P < 0.001, **P < 0.01. (**D**) HE staining of representative lung sections from each indicated group of treated mice. Scale bar, 100 μm. (**E**) Lung inflammation score of the HE staining of lung sections from each indicated group of treated mice (non-treated *n* = 8, ALI_PBS *n* = 12, ALI_HBEC-EVs *n* = 12). ****P < 0.0001, **P < 0.01. (**F**) IL-6 and TNF-α protein concentration determined by ELISA in the BALF from each indicated group of treated mice (non-treated *n* = 8, ALI_PBS *n* = 7, ALI_HBEC-EVs *n* = 10). ****P < 0.0001, **P < 0.01, *P < 0.05. (**G**) Immunohistochemical staining of FPR2 in representative lung sections from the non-treated group and mouse model of ALI induced by both LPS and poly (I:C). Green arrows indicate FPR2-positive macrophage congregation, whereas red arrows indicate FPR2-positive alveolar epithelial cells. Scale bar, 100 μm. (**H**) Western blots showing the levels of p-NF-κB, NF-κB, and β-actin in the lungs of mice from each indicated group treatment group. (**I**) Schematic diagram of the proposed mechanism of the therapeutic potential of HBEC-EVs against ALI pathologies.

In this study, we employed a mouse model of ALI induced by both LPS and poly (I:C) to investigate the physiological immunomodulatory effects of intratracheal HBEC-EV administration during and after the onset of lung inflammation (Fig. 5A). Mice treated with HBEC-EVs showed visibly reduced BAL hemorrhage compared with the PBS control group, indicating a lower degree of lung injury following HBEC-EV treatment (Fig. 5B). Furthermore, HBEC-EVs significantly decreased the total number of BAL cells and neutrophils compared with the PBS control group (Fig. 5C). HE staining of lung tissues demonstrated that both LPS-and poly (I:C)-induced lung injury were alleviated by HBEC-EVs (Fig. 5D). HBEC-EV treatment also led to a significant reduction in peribronchial and perivascular inflammation (Fig. 5E). We employed ELISA to measure the levels of inflammatory cytokines, and found that, in comparison with PBS the control group, the levels of IL-6 and TNF-α were significantly reduced in the BALF of the HBEC-EV treatment group (Fig. 5F). Additionally, we assessed the effects of HBEC-EVs on FPR-NF-κB signaling in the lungs of ALI mice. Our analysis confirmed the presence of FPR2-positive alveolar epithelial cells and macrophage congregation in both LPS-and poly (I:C)-induced lung injury models (Fig. 5G). Consistently, HBEC-EV treatment significantly suppressed the expression of p-NF-κB in the mouse lungs compared with the PBS control group (Fig. 5H). Collectively, these findings provide evidence that intratracheal HBEC-EV administration can alleviate acute lung inflammation through FPR-NF-κB signaling through cargo transfer. HBEC-EVs represent a potential therapeutic strategy for ALI through immunomodulation (Fig. 5I).

## Discussion

In this study, we investigated the therapeutic potential of HBEC-EVs for treating ALI. HBEC-EVs, isolated from lung bronchial epithelial cells, demonstrated significant immunomodulatory effects in cellular models of ALI by mitigating pro-inflammatory responses. These effects were can be partly attributed to nine of the top ten miRNAs enriched in HBEC-EVs, which regulate immune-related pathways. Additionally, our proteomic analysis unveiled the presence of proteins within HBEC-EVs that participate in WNT and NF-κB signaling pathways, critical regulators of inflammation. Notably, ANXA1, a protein within HBEC-EVs, interacts with FPR2 receptors, leading to anti-inflammatory effects through the suppression of NF-κB signaling in inflamed epithelial cells, including ATII cells. Our findings were further supported by an ALI mouse model, where the intratracheal administration of HBEC-EVs resulted in decreased lung injury, inflammatory cell infiltration, and cytokine levels. Collectively, these findings suggest the involvement of the NF-κB signaling pathway in the mechanisms underlying HBEC-EV-mediated immunomodulation via miRNAs and ANXA1 cargo.

Recent studies have highlighted the promise of both MSCs and MSC-EVs as agents for ALI treatment. These cells possess anti-inflammatory properties, support tissue repair, and modulate the immune response^30^. Furthermore, EVs contain bioactive molecules with anti-inflammatory and regenerative benefits^12^, making them potential targeted therapeutic options. However, ongoing research is essential to address safety concerns and optimize treatment protocols for improved outcomes. For clinical applications, ruling out the possibility of MSCs serving as a source of abnormal fibroblasts or promoting cancer progression^31^. In our study, we focused on normal lung epithelial cells as a source of EVs and elucidated their immunomodulatory functions in lung pathology. Lung epithelial cells line the airways, produce mucus, facilitate mucus clearance through ciliary movement, and indirectly support gas exchange. EVs play a crucial role as messengers for intercellular communication, contributing to the maintenance of a healthy lung microenvironment^14^. Our findings suggest that HBEC-EVs can be transferred to both epithelial cells and alveolar macrophages, potentially regulating immunity, inflammation, and facilitating lung tissue repair.

ANXA1 is a key player in the regulation of inflammatory and immune responses^32^. It exerts its anti-inflammation by binding to specific cell receptors, such as FPR2, and inhibiting the activation of pro-inflammatory pathways, particularly NF-kB signaling. This regulatory role makes ANXA1 essential for maintaining immune balance and tissue protection. Previous studies have shown that EVs contain ANXA1^33,34^. For instance, Leoni et al. reported that intestinal epithelial cells release the potent endogenous pro-resolving mediator ANXA1 as a component of EVs, promoting intestinal mucosal wound repair^33^. Together with our data, these findings suggest that epithelial cell-derived EVs may hold therapeutic potential for mitigating donor organ injuries and maintaining tissue homeostasis in the microenvironment.

Our study has several limitations. First, we utilized a mouse model of ALI induced by both LPS and poly (I:C). Although this model offers valuable insights into the response to multiple inflammatory stimuli, it may not fully replicate the complex pathophysiology of human ALI. Therefore, caution is warranted when extrapolating these findings to human ALI because of the inherent limitations of the model and its artificial, nonphysiological nature. The second limitation pertains to miRNA-mediated immunomodulation. We identified nine of the top 10 miRNAs enriched in HBEC-EVs; however, the precise mechanisms through which these miRNAs regulate immune responses, particularly in the context of TLR-NF-κB signaling, require further elucidation. In addition, it is important to consider the potential differences in miRNA downregulation between humans and mice. Nevertheless, many of the miRNAs abundant in HBEC-EVs have already been reported for NF-κB pathway inhibition by conserved miRNA targets (Supplementary Table 1, Supplementary Fig. 1). This suggests that despite potential interspecies variations, there is a significant overlap in miRNA-mediated regulation of the NF-κB pathway in both humans and mice. Third, although we demonstrated EV ANXA1 interacting with FPR2 and its role in suppressing NF-κB signaling in the inflamed epithelium, this interaction was not investigated in THP-1 cells. In vitro cellular models, such as PMA-induced THP-1 macrophages and HBECs, offer controlled environments but may not fully recapitulate the complex interactions occurring in the lung microenvironment during ALI. Interactions with other immune cell types in the lungs could play a crucial role in the development of ALI. These limitations underscore the need for future research to comprehensively understand the potential of HBEC-EVs as a therapeutic option for ALI.

In conclusion, our study has provided valuable insights into the potential therapeutic role of HBEC-EVs in the treatment of ALI. These vesicles exhibit immunomodulatory properties through miRNA transfer and ANXA1 presence, which can suppress NF-κB signaling and reduce inflammation. Although further research is necessary to fully elucidate these mechanisms, HBEC-EVs hold promise as a novel approach for mitigating lung injury and inflammation in ALI.

### Supplementary information

Supporting Information

Reporting Summary

## Data Availability

The source data for the graphs in this study will be provided in “10.6084/m9.figshare.25458145.v1”. The Western blot uncropped images are provided in Supplementary Fig. 4 in Supplementary Information. The miRNA-seq analysis was deposited in the GEO database (GSE156572)^14^. All other supporting data generated and/or analyzed during this study are available from the corresponding author upon reasonable request.

## References

[1] Rubenfeld GD (2005). Incidence and outcomes of acute lung injury. N. Engl. J. Med.

[2] Bhatia M, Zemans RL, Jeyaseelan S (2012). Role of chemokines in the pathogenesis of acute lung injury. Am. J. Respir. Cell Mol. Biol..

[3] Wang P (2020). A cross-talk between epithelium and endothelium mediates human alveolar-capillary injury during SARS-CoV-2 infection. Cell Death Dis..

[4] Mu S (2018). Unfractionated heparin ameliorates pulmonary microvascular endothelial barrier dysfunction via microtubule stabilization in acute lung injury. Respir. Res.

[5] Stavely R, Nurgali K (2020). The emerging antioxidant paradigm of mesenchymal stem cell therapy. Stem Cells Transl. Med.

[6] Matthay MA (2010). Therapeutic potential of mesenchymal stem cells for severe acute lung injury. Chest.

[7] Olajuyin AM, Zhang X, Ji HL (2019). Alveolar type 2 progenitor cells for lung injury repair. Cell Death Discov..

[8] Wang D, Morales JE, Calame DG, Alcorn JL, Wetsel RA (2010). Transplantation of human embryonic stem cell-derived alveolar epithelial type II cells abrogates acute lung injury in mice. Mol. Ther..

[9] Tieu A (2020). An analysis of mesenchymal stem cell-derived extracellular vesicles for preclinical use. ACS Nano.

[10] Tkach M, Thery C (2016). Communication by extracellular vesicles: where we are and where we need to go. Cell.

[11] Fujita Y, Kosaka N, Araya J, Kuwano K, Ochiya T (2015). Extracellular vesicles in lung microenvironment and pathogenesis. Trends Mol. Med.

[12] Fujita Y, Kadota T, Araya J, Ochiya T, Kuwano K (2018). Clinical application of mesenchymal stem cell-derived extracellular vesicle-based therapeutics for inflammatory lung diseases. J. Clin. Med.

[13] Fujita Y (2015). Suppression of autophagy by extracellular vesicles promotes myofibroblast differentiation in COPD pathogenesis. J. Extracell. Vesicles.

[14] Kadota T (2021). Human bronchial epithelial cell-derived extracellular vesicle therapy for pulmonary fibrosis via inhibition of TGF-beta-WNT crosstalk. J. Extracell. Vesicles.

[15] Gupta R (2019). Intercellular communication between airway epithelial cells is mediated by exosome-like vesicles. Am. J. Respir. Cell Mol. Biol..

[16] Araya J (2007). Squamous metaplasia amplifies pathologic epithelial-mesenchymal interactions in COPD patients. J. Clin. Invest..

[17] Konishi S, Tata A, Tata PR (2022). Defined conditions for long-term expansion of murine and human alveolar epithelial stem cells in three-dimensional cultures. STAR Protoc..

[18] Yoshioka, Y. et al. Comparative marker analysis of extracellular vesicles in different human cancer types. *J. Extracell. Vesicles* **2**, 10.3402/jev.v2i0.20424 (2013). 10.3402/jev.v2i0.20424 PMC3760642 24009892

[19] Sun YQ (2012). Human pluripotent stem cell-derived mesenchymal stem cells prevent allergic airway inflammation in mice. Stem Cells.

[20] Vlachos IS (2015). DIANA-miRPath v3.0: deciphering microRNA function with experimental support. Nucleic Acids Res..

[21] Thery C (2018). Minimal information for studies of extracellular vesicles 2018 (MISEV2018): a position statement of the International Society for Extracellular Vesicles and update of the MISEV2014 guidelines. J. Extracell. Vesicles.

[22] Matthay MA (2019). Acute respiratory distress syndrome. Nat. Rev. Dis. Prim..

[23] Thompson BT, Chambers RC, Liu KD (2017). Acute respiratory distress syndrome. N. Engl. J. Med..

[24] O’Brien K, Breyne K, Ughetto S, Laurent LC, Breakefield XO (2020). RNA delivery by extracellular vesicles in mammalian cells and its applications. Nat. Rev. Mol. Cell Biol..

[25] Ramilowski JA (2015). A draft network of ligand-receptor-mediated multicellular signalling in human. Nat. Commun..

[26] Zhang, K. et al. Upregulated gga-miR-16-5p Inhibits the proliferation cycle and promotes the apoptosis of MG-Infected DF-1 cells by repressing PIK3R1-mediated the PI3K/Akt/NF-kappaB pathway to exert anti-inflammatory effect. *Int. J. Mol. Sci.* **20**, 1036 (2019). 10.3390/ijms20051036 PMC6429190 30818821

[27] Yang YH, Morand E, Leech M (2013). Annexin A1: potential for glucocorticoid sparing in RA. Nat. Rev. Rheumatol..

[28] Ye RD (2009). International union of basic and clinical Pharmacology. LXXIII. Nomenclature for the formyl peptide receptor (FPR) family. Pharm. Rev..

[29] Gavins FN, Hickey MJ (2012). Annexin A1 and the regulation of innate and adaptive immunity. Front. Immunol..

[30] Lee JW, Fang X, Krasnodembskaya A, Howard JP, Matthay MA (2011). Concise review: Mesenchymal stem cells for acute lung injury: role of paracrine soluble factors. Stem Cells.

[31] Glassberg MK (2017). Allogeneic human mesenchymal stem cells in patients with idiopathic pulmonary fibrosis via intravenous delivery (AETHER): A Phase I safety clinical trial. Chest.

[32] Oliveira LG (2017). Annexin A1 Is involved in the resolution of inflammatory responses during Leishmania braziliensis infection. J. Immunol..

[33] Leoni G (2015). Annexin A1-containing extracellular vesicles and polymeric nanoparticles promote epithelial wound repair. J. Clin. Invest..

[34] Li Q, Liu W, Wang Z, Wang C, Ai Z (2021). Exosomal ANXA1 derived from thyroid cancer cells is associated with malignant transformation of human thyroid follicular epithelial cells by promoting cell proliferation. Int. J. Oncol..

