# Supplementary material for: Mitigation of acute lung injury by human bronchial epithelial cell-derived extracellular vesicles via ANXA1-mediated FPR signaling
Source: Commun Biol. 2024 May 6;7:514. doi: 10.1038/s42003-024-06197-3 (PMC11074269; doi:10.1038/s42003-024-06197-3)
Supplement: Supplementary file 1 — Supporting Information [file 42003_2024_6197_MOESM1_ESM.pdf]

Supplementary Information

Mitigation of Acute Lung Injury by Human Bronchial Epithelial Cell-derived Extracellular Vesicles via ANXA1-mediated FPR Signaling

Yu Fujita[1,2,3+*], Tsukasa Kadota[1+], Reika Kaneko[2], Yuta Hirano[1], Shota Fujimoto[1], Naoaki Watanabe[1], Ryusuke Kizawa[1,2], Takashi Ohtsuka[4], Kazuyoshi Kuwano[1], Takahiro Ochiya[5], Jun Araya[1]

[1]Division of Respiratory Diseases, Department of Internal Medicine, The Jikei University School of Medicine, Tokyo, Japan.

[2]Division of Next-Generation Drug Development, Research Center for Medical Sciences, The Jikei University School of Medicine, Tokyo, Japan.

[3] Center for Exosome Medical Research, The Jikei University School of Medicine, Tokyo, Japan.

[4] Division of Thoracic Surgery, Department of Surgery, The Jikei University School of Medicine, Tokyo, Japan.

[5] Department of Molecular and Cellular Medicine, Institute of Medical Science, Tokyo Medical University, Tokyo, Japan.

[+]These authors equally contributed to this work.

Supplementary Figure 1

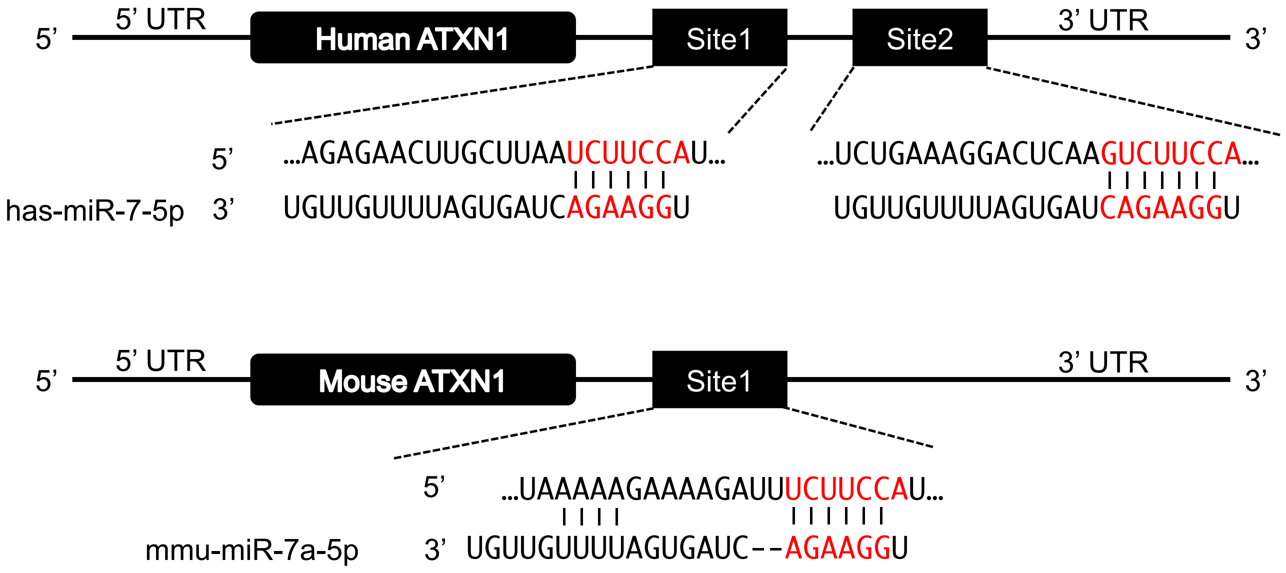

Supplementary Figure 1: A schematic representation of has-miR-7-5p and mmu-miR-7A-5p–binding sites in the human and mouse 3'UTR of ATXN1 mRNA.

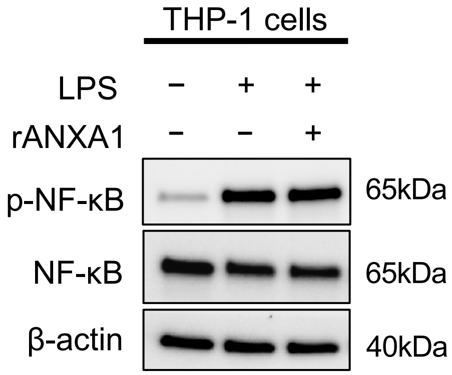

Supplementary Figure 2: Western blots showing the amount of p-NF-κB, NF-κB, and β-actin in THP-1 cells treated with recombinant ANXA1 (rANXA1) in the presence of LPS (1 μg/ml). THP-1 cells were treated with 100 nM PMA for 24 h before the experiments.

Supplementary Figure 3

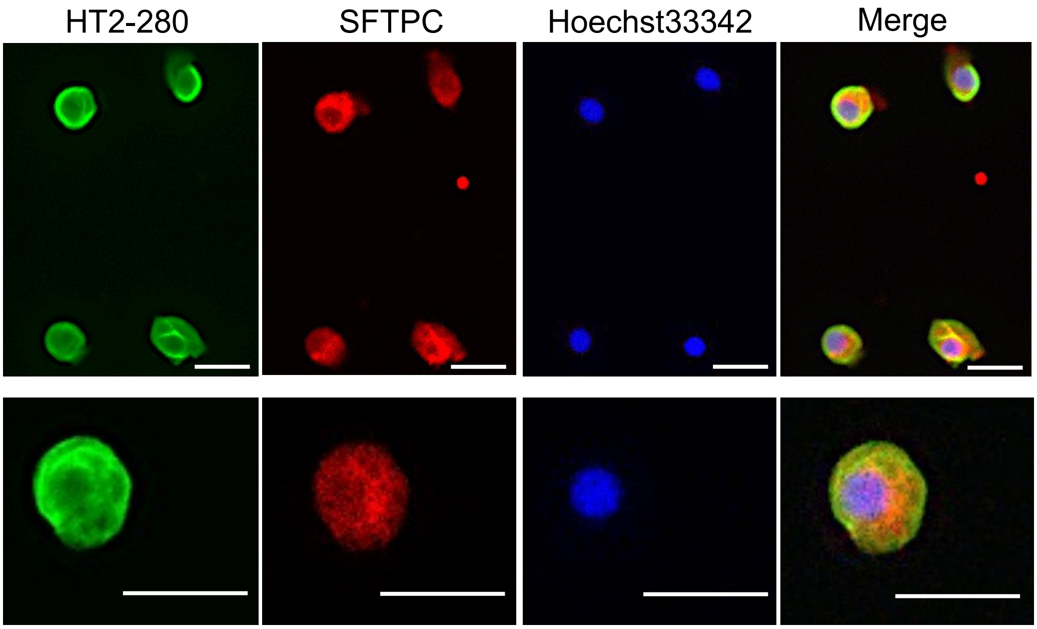

Supplementary Figure 3: HT2-280 and SFTPC Expression of type II alveolar epithelial cells (ATII) markers by immunofluorescence. Scale bar 20μm.

Supplementary Figure 4

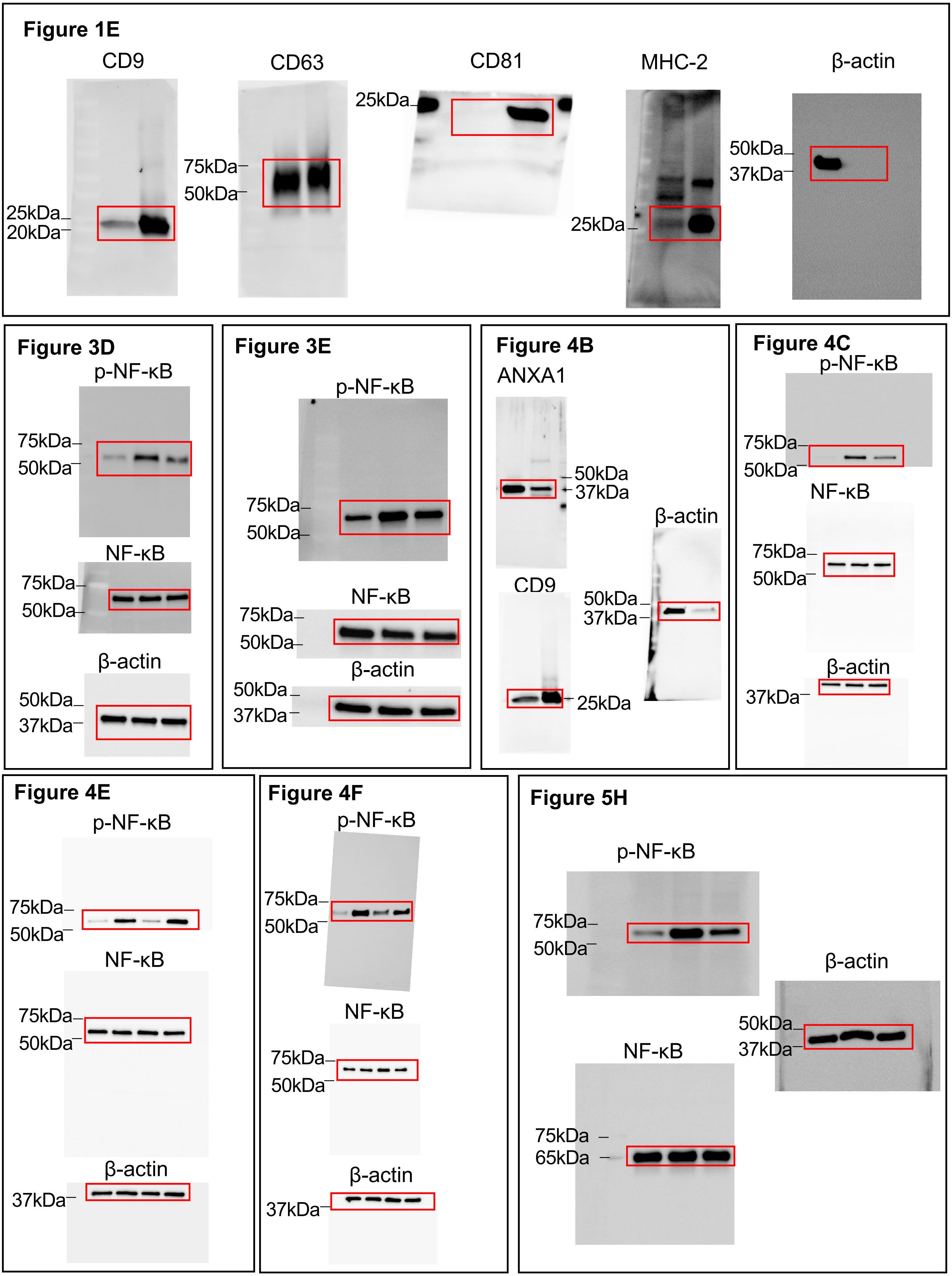

Supplementary Figure 4: Unedited/uncropped western blots for main figures

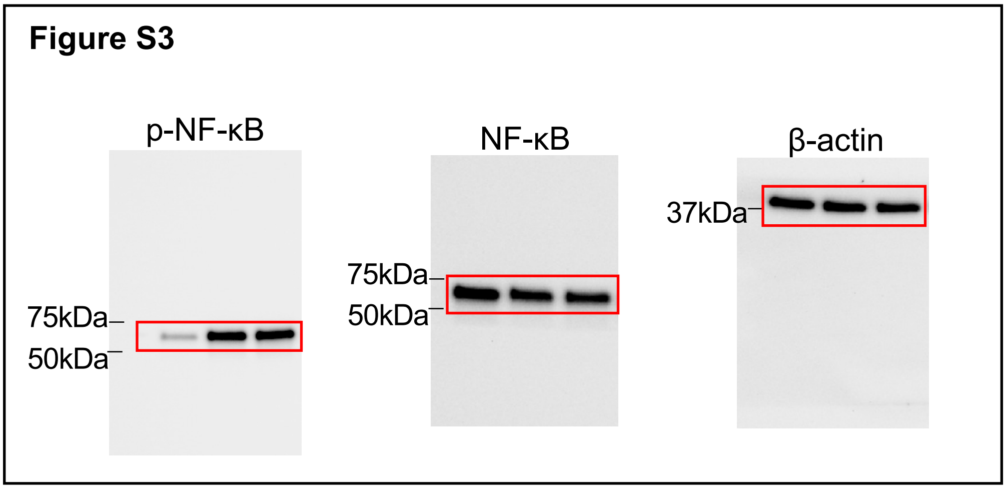

Supplementary Figure 4 (continue): Unedited/uncropped western blots for main figures

Supplementary Table 1. Top 10 miRNAs directly targeting NF-κB pathway-related genes in humans and mice

	Human	Mouse	Sequence	Direct target NF-κB pathway-related genes (downregulation) both in human and mouse	miRNA family conservation by TargetScan	
	miRNA name<br>Accession	miRNA name<br>Accession			Family name	Conservation
1	hsa-miR-7-5p<br>MIMAT0000252	mmu-miR-7a-5p<br>MIMAT0000677	UGGAAGACUAGUGAUUUUGUUGU	ATXN1[1]	miR-7-5p	broadly conserved
2	has-miR-125b-5p<br>MIMAT0000423	mmu-miR-125b-5p<br>MIMAT0000136	UCCCUGAGACCCUAACUUGUGA	A20[2], TRAF6[3,4]	miR-125-5p/351-5p	broadly conserved
3	hsa-let-7a-5p<br>MIMAT0000062	mmu-let-7a-5p<br>MIMAT0000521	UGAGGUAGUAGGUUGUAUAGUU	SOCS1[5], Endothelin-1[6]	let-7-5p/miR-98-5p	broadly conserved
4	has-miR-125a-5p<br>MIMAT0000443	mmu-miR-125a-5p<br>MIMAT0000135	ACAGGUGAGGUUCUUGGGAGCC	A20[2], TRAF6[3,4]	miR-125-5p/351-5p	broadly conserved
5	has-let-7b-5p<br>MIMAT0000063	mmu-let-7b-5p<br>MIMAT0000522	UGAGGUAGUAGGUUGUGUGGUU	HMGA2[7]	let-7-5p/miR-98-5p	broadly conserved
6	has-let-7f-5p<br>MIMAT0000067	mmu-let-7f-5p<br>MIMAT0000525	UGAGGUAGUAGAUUGUAUAGUU	IL-10[8], IGF-1[9]	let-7-5p/miR-98-5p	broadly conserved
7	has-let-7i-5p<br>MIMAT0000415	mmu-let-7i-5p<br>MIMAT0000122	UGAGGUAGUAGUUUGUGCUGUU	IL-6[10]	let-7-5p/miR-98-5p	broadly conserved
8	has-miR-26a-5p<br>MIMAT0000082	mmu-miR-26a-5p<br>MIMAT0000533	UUCAAGUAAUCCAGGAUAGGCU	CHAC1[11], IL6[12]	miR-26-5p	broadly conserved
9	has-miR-16-5p<br>MIMAT0000069	mmu-miR-16-5p<br>MIMAT0000527	UAGCAGCACGUAAAUAUUGGCG	PIK3R1[13]	miR-15-5p/16-5p/195-5p<br>/322-5p/497-5p	broadly conserved
10	has-let-7c-5p<br>MIMAT0000064	mmu-let-7c-5p<br>MIMAT0000523	UGAGGUAGUAGGUUGUAUGGUU	DMP-1[14]	let-7-5p/miR-98-5p	broadly conserved

Supplementary Table 2. Primer information

Gene symbol	Name	Forward	Reverse
ACTB	actin beta	CATGTACGTTGCTATCCAGGC	CTCCTTAATGTCACGCACGAT
IL6	interleukin 6	ACTCACCTCTTCAGAACGAATTG	CCATCTTTGGAAGGTTCAGGTTG
IL8	C-X-C motif chemokine ligand 8	TTTTGCCAAGGAGTGCTAAAGA	AACCCTCTGCACCCAGTTTTC
TNFα	tumor necrosis factor alpha	CCCAGGGACCTCTCTCTAATC	ATGGGCTACAGGCTTGTCACT
FPR1	formyl peptide receptor 1	TGGGAGGACATTGGCCTTTC	GGATGCAGGACGCAAACAC
FPR2	formyl peptide receptor 2	AGTCTGCTGGCTACACTGTTC	TGGTAATGTGGCCGTGAAAGA
FPR3	formyl peptide receptor 3	GCTAGTCCACGGAGTCACCT	GGTAGGATGGCACTGAAAGAGA

Supplementary References

1. Lou, L.Q., Zhou, W.Q., Song, X. & Chen, Z. Elevation of hsa-miR-7-5p level mediated by CtBP1-p300-AP1 complex targets ATXN1 to trigger NF-kappaB-dependent inflammation response. *J Mol Med (Berl)* **101**, 223-235 (2023).
2. Kim, S.-W., *et al.* MicroRNAs miR-125a and miR-125b constitutively activate the NF-κB pathway by targeting the tumor necrosis factor alpha-induced protein 3 (TNFAIP3, A20). *Proceedings of the National Academy of Sciences* **109**, 7865-7870 (2012).
3. Rasheed, Z., Rasheed, N., Abdulmonem, W.A. & Khan, M.I. MicroRNA-125b-5p regulates IL-1beta induced inflammatory genes via targeting TRAF6-mediated MAPKs and NF-kappaB signaling in human osteoarthritic chondrocytes. *Sci Rep* **9**, 6882 (2019).
4. Wang, W. & Guo, Z.-H. Downregulation of lncRNA NEAT1 Ameliorates LPS-Induced Inflammatory Responses by Promoting Macrophage M2 Polarization via miR-125a-5p/TRAF6/TAK1 Axis. *Inflammation* **43**, 1548-1560 (2020).
5. Yan, C., *et al.* Csi-let-7a-5p delivered by extracellular vesicles from a liver fluke activates M1-like macrophages and exacerbates biliary injuries. *Proc Natl Acad Sci U S A* **118**(2021).
6. Thakur, D., *et al.* Inhibition of nuclear factor kappaB in the lungs protect bleomycin-induced lung fibrosis in mice. *Mol Biol Rep* **49**, 3481-3490 (2022).
7. Hong, Y., *et al.* High-frequency repetitive transcranial magnetic stimulation (rTMS) protects against ischemic stroke by inhibiting M1 microglia polarization through let-7b-5p/HMGA2/NF-kappaB signaling pathway. *BMC Neurosci* **23**, 49 (2022).
8. Gao, X.R., *et al.* NF-kappaB/let-7f-5p/IL-10 pathway involves in wear particle-induced osteolysis by inducing M1 macrophage polarization. *Cell Cycle* **17**, 2134-2145 (2018).
9. Li, Y., *et al.* IL-1beta/NF-kappaB signaling inhibits IGF-1 production via let-7f-5p in dendritic epidermal T cells. *J Leukoc Biol* **112**, 1677-1690 (2022).
10. Xie, K., *et al.* lnc001776 Affects CPB2 Toxin-Induced Excessive Injury of Porcine Intestinal Epithelial Cells via Activating JNK/NF-kB Pathway through ssc-let-7i-5p/IL-6 Axis. *Cells* **12**(2023).
11. Li, S., *et al.* Inhibiting Rab27a in renal tubular epithelial cells attenuates the inflammation of diabetic kidney disease through the miR-26a-5p/CHAC1/NF-kB pathway. *Life Sci* **261**, 118347 (2020).
12. Chen, Y., Zhou, X. & Wu, Y. The miR-26a-5p/IL-6 axis alleviates sepsis-induced acute kidney injury by inhibiting renal inflammation. *Renal Failure* **44**, 551-561 (2022).
13. Zhang, K., *et al.* Upregulated gga-miR-16-5p Inhibits the Proliferation Cycle and Promotes the Apoptosis of MG-Infected DF-1 Cells by Repressing PIK3R1-Mediated the PI3K/Akt/NF-kappaB Pathway to Exert Anti-Inflammatory Effect. *Int J Mol Sci* **20**(2019).
14. Yuan, H., *et al.* MicroRNA let-7c-5p Suppressed Lipopolysaccharide-Induced Dental Pulp Inflammation by Inhibiting Dentin Matrix Protein-1-Mediated Nuclear Factor kappa B (NF-kappaB) Pathway In Vitro and In Vivo. *Med Sci Monit* **24**, 6656-6665 (2018).
