# Supplementary material for: Mitigation of acute lung injury by human bronchial epithelial cell-derived extracellular vesicles via ANXA1-mediated FPR signaling
Source: Commun Biol. 2024 May 6;7:514. doi: 10.1038/s42003-024-06197-3 (PMC11074269; doi:10.1038/s42003-024-06197-3)
Supplement: Supplementary file 2 — Reporting Summary [file 42003_2024_6197_MOESM2_ESM.pdf]

nature portfolio

Corresponding author(s): Yu Fujita

Last updated by author(s): 2024/03/22

Reporting Summary

Nature Portfolio wishes to improve the reproducibility of the work that we publish. This form provides structure for consistency and transparency in reporting. For further information on Nature Portfolio policies, see our Editorial Policies and the Editorial Policy Checklist.

Statistics

For all statistical analyses, confirm that the following items are present in the figure legend, table legend, main text, or Methods section.

n/a	Confirmed	
☐	☒	The exact sample size (n) for each experimental group/condition, given as a discrete number and unit of measurement
☐	☒	A statement on whether measurements were taken from distinct samples or whether the same sample was measured repeatedly
☐	☒	The statistical test(s) used AND whether they are one- or two-sided<br>*Only common tests should be described solely by name; describe more complex techniques in the Methods section.*
☐	☒	A description of all covariates tested
☐	☒	A description of any assumptions or corrections, such as tests of normality and adjustment for multiple comparisons
☐	☒	A full description of the statistical parameters including central tendency (e.g. means) or other basic estimates (e.g. regression coefficient) AND variation (e.g. standard deviation) or associated estimates of uncertainty (e.g. confidence intervals)
☐	☒	For null hypothesis testing, the test statistic (e.g. F, t, r) with confidence intervals, effect sizes, degrees of freedom and P value noted<br>*Give P values as exact values whenever suitable.*
☒	☐	For Bayesian analysis, information on the choice of priors and Markov chain Monte Carlo settings
☒	☐	For hierarchical and complex designs, identification of the appropriate level for tests and full reporting of outcomes
☐	☒	Estimates of effect sizes (e.g. Cohen's d, Pearson's r), indicating how they were calculated

Our web collection on statistics for biologists contains articles on many of the points above.

Software and code

Policy information about availability of computer code

Data collection	GSE156572
Data analysis	Prism version 9 (GraphPad Software, San Diego, CA, USA) was utilized for statistical analysis.

For manuscripts utilizing custom algorithms or software that are central to the research but not yet described in published literature, software must be made available to editors and reviewers. We strongly encourage code deposition in a community repository (e.g. GitHub). See the Nature Portfolio guidelines for submitting code & software for further information.

Data

Policy information about availability of data

All manuscripts must include a data availability statement. This statement should provide the following information, where applicable:

- Accession codes, unique identifiers, or web links for publicly available datasets
- A description of any restrictions on data availability
- For clinical datasets or third party data, please ensure that the statement adheres to our policy

Web links for publicly available datasets

Research involving human participants, their data, or biological material

Policy information about studies with human participants or human data. See also policy information about sex, gender (identity/presentation), and sexual orientation and race, ethnicity and racism.

Reporting on sex and gender	Airway tissues were obtained from pneumonectomy and lobectomy specimens of patients with lung cancer at The Jikei University School of Medicine.
Reporting on race, ethnicity, or other socially relevant groupings	The specimens are all from Japanese.
Population characteristics	We describe the population characteristics in the Method section.
Recruitment	Airway tissues were obtained from pneumonectomy and lobectomy specimens of patients with lung cancer at The Jikei University School of Medicine.
Ethics oversight	Informed consent was obtained from all surgical participants as part of an ongoing research protocol approved by the Ethical Committee of Jikei University School of Medicine (#20-153 (5443)).

Note that full information on the approval of the study protocol must also be provided in the manuscript.

Field-specific reporting

Please select the one below that is the best fit for your research. If you are not sure, read the appropriate sections before making your selection.

☒ Life sciences ☐ Behavioural & social sciences ☐ Ecological, evolutionary & environmental sciences

For a reference copy of the document with all sections, see nature.com/documents/nr-reporting-summary-flat.pdf

Life sciences study design

All studies must disclose on these points even when the disclosure is negative.

Sample size	We cultured and used bronchial epithelial cells for each experiments (N=3).
Data exclusions	The participants lacked a history of pulmonary fibrosis, COPD or any other inflammatory diseases.
Replication	We cultured and used bronchial epithelial cells for each experiments (N=3).
Randomization	No randomization
Blinding	No blinding

Reporting for specific materials, systems and methods

We require information from authors about some types of materials, experimental systems and methods used in many studies. Here, indicate whether each material, system or method listed is relevant to your study. If you are not sure if a list item applies to your research, read the appropriate section before selecting a response.

Materials & experimental systems

n/a	Involved in the study	
☐	☒	Antibodies
☒	☐	Eukaryotic cell lines
☒	☐	Palaeontology and archaeology
☐	☒	Animals and other organisms
☒	☐	Clinical data
☒	☐	Dual use research of concern
☒	☐	Plants

Methods

n/a	Involved in the study	
☒	☐	ChIP-seq
☒	☐	Flow cytometry
☒	☐	MRI-based neuroimaging

Antibodies

Antibodies used	mouse anti-CD9 (Santa Cruz Biotechnology, sc-59140), mouse anti-CD63 (BD Pharmingen, 556019), mouse anti-CD81 (Santa Cruz Biotechnology, sc-555675), mouse anti-β-actin (Millipore, MAB1501), mouse anti-MHC-2 (Cell Signaling Technology, 68258), mouse

	anti–NK-κB (Cell Signaling Technology, 8242), mouse anti–phosphor-NK-κB (Cell Signaling Technology, 3033), rabbit anti-ANXA1 (Thermo Fisher Scientific, 71-3400), and rabbit anti-FPR2 (Novusbio, NLS1878).
Validation	All antibodies were validated by western blotting.

Animals and other research organisms

Policy information about studies involving animals; ARRIVE guidelines recommended for reporting animal research, and Sex and Gender in Research

Laboratory animals	Male mice aged 6–7 weeks C57BL/6J (Charles River Laboratories) were employed in the experiments.
Wild animals	Animal experiments were performed in compliance with the guidelines of the Institute for Laboratory Animal Research.
Reporting on sex	Male mice aged 6–7 weeks C57BL/6J
Field-collected samples	Briefly, LPS was intraperitoneally (i.p.) injected at a dose of 5 μg/mg in 50 μl PBS. Four hours following LPS injection, poly (I:C) at a dose of 5 μg/mg in 50 μl PBS and HBEC-EVs (2×109 particles)/PBS control was intratracheally (i.t.) injected. After 24 h of induction, the mice were euthanized. Lung tissue sections were stained with hematoxylin-eosin (HE). The lung inflammation score was calculated using a previously reported method 19. BALF was collected by cannulating the trachea with a 20-gauge shielded intravenous catheter, instilling 0.8 ml of sterile PBS four times, and collecting the fluid by gentle aspiration. The BALF was centrifuged for 5 min at 400×g on microscopic slides, and the BALF cells were stained using the Diff-Quick method. The resulting fluid was passed through a 0.45-μm filter and either used immediately or stored at -70? for subsequent measurements of IL-6 and TNF-α levels by ELISA (R&D Systems).
Ethics oversight	All animal experiments were performed in compliance with the guidelines of the Institute for Laboratory Animal Research, Jikei University School of Medicine (Number: 2018–071).

Note that full information on the approval of the study protocol must also be provided in the manuscript.

Plants

Seed stocks	NA
Novel plant genotypes	NA
Authentication	NA
